# Association of cytokines levels, psychopathology and cognition among CR-TRS patients with metabolic syndrome

**DOI:** 10.1038/s41537-024-00469-x

**Published:** 2024-04-16

**Authors:** Yeqing Dong, Minghuan Zhu, Yanzhe Li, Nannan Liu, Xinxu Wang, Bing Yang, Shen Li, Zezhi Li

**Affiliations:** 1 Institute of Mental Health, Tianjin Anding Hospital, Mental Health Center of Tianjin Medical University, Tianjin, 300222 China; 2 Psychoneuromodulation Center, Tianjin Anding Hospital, Mental Health Center of Tianjin Medical University, Tianjin, 300222 China; 3 Clinical Research Center for Mental Disorders, Shanghai Pudong New Area Mental Health Center, School of Medicine, Tongji University, Shanghai, 200124 China; 4 Department of Cell Biology, College of Basic Medical Sciences, Tianjin Medical University, Tianjin, 300070 China; 5 Department of Nutritional and Metabolic Psychiatry, the Affiliated Brain Hospital of Guangzhou Medical University, Guangzhou, 510370 China; 6 Guangdong Engineering Technology Research Center for Translational Medicine of Mental Disorders, Guangzhou, China; 7 Key Laboratory of Neurogenetics and Channelopathies of Guangdong Province and the Ministry of Education of China, Guangzhou Medical University, Guangzhou, China; 8 Jiangsu Key Laboratory of Neurodegeneration, Nanjing Medical University, Nanjing, China

**Keywords:** Biomarkers, Schizophrenia

## Abstract

Clozapine-resistant treatment-refractory schizophrenia (CR-TRS) patients face significant clinical challenges. While links between metabolic syndrome (MetS) and inflammatory cytokines in schizophrenia have been established, the relationship between MetS and cytokine levels in CR-TRS patients remains unexplored. This study aimed to investigate the relationship between cytokines levels, clinical symptoms and cognitive impairments in CR-TRS patients, both with and without MetS. The study included 69 CR-TRS patients (31with MetS and 38 without MetS) and 84 healthy controls. The levels of IL-2, IL-6, TNF-α and routine biochemical parameters were measured. Psychopathological symptoms and cognitive function were assessed using the Positive and Negative Syndrome Scale (PANSS) and the Repeatable Battery for the Assessment of Neuropsychological Status (RBANS), respectively. We found that CR-TRS patients with MetS displayed lower cognitive function scores compared to those without MetS, even when accounting for potential confounders. TNF-α levels were significantly higher in CRTRS patients with MetS compared to those without MetS, demonstrating substantial pathophysiological potential for CR-TRS patients with MetS via receiver operating characteristic curve (ROC). In CR-TRS patients without MetS, IL-2 independently contributed to the total score and general psychopathology subscore of PANSS. Additionally, IL-6 exhibited an independent contribution to the positive subscore of PANSS. In terms of cognition function, IL-6 independently contributed to the delayed memory of RBANS in CR-TRS patients without MetS. TNF-α could potentially serve as a predictive marker for distinguishing between CR-TRS patients with/without MetS, while IL-2 and IL-6 could independently contribute to psychopathological symptoms or cognitive function in CRTRS patients without MetS. Our study provided insights into the potential interplay between cytokines, clinical symptoms and cognitive impairments in CR-TRS patients with/without MetS.

## Introduction

Schizophrenia is a chronic psychiatric disorder characterized by the presence of positive symptoms, negative symptoms and cognitive dysfunctions^1^. While pharmacological treatments remain a primary approach for managing schizophrenia, their application can vary and be inconsistent. Currently, about 30% patients with schizophrenia continue to experience poor outcomes even after undergoing treatment with two or more antipsychotics, a group known as “treatment-resistant schizophrenia (TRS)” or “treatment-refractory schizophrenia”^2,3^. There is a general consensus that clozapine is considered the preferred treatment for TRS individuals^4^. However, about 40%-70% of TRS patients do not experience substantial improvement even after receiving adequate clozapine therapy, leading to their classification as having clozapine-resistant treatment-refractory schizophrenia (CR-TRS)^5,6^. Compared to TRS patients, CR-TRS patients tend to exhibit heightened severity in both positive and negative symptoms, experience more frequent relapses and hospitalizations^7,8^.

Metabolic syndrome (MetS) typically refers to a cluster of metabolic alterations that collectively increased the risk of cardiovascular disease^9^. MetS is characterized by the simultaneous presence of at least three interrelated cardiovascular risk factors, including abdominal obesity, hyperglycemia, hypertension, high triglycerides or low high-density lipoprotein cholesterol levels^10^. Convincing evidence suggests that individuals with schizophrenia face an elevated risk of developing MetS^11^, a condition known to have substantial implications for future morbidity and mortality rates. A systematic review and meta-analysis have indicated that the prevalence of MetS in individuals with schizophrenia is approximately 32.5%, with the highest rates observed among individuals prescribed clozapine (51.9%)^12^. In addition, cognitive impairment is recognized as another hallmark of schizophrenia^13^, and some studies have demonstrated that MetS and its components are significant risk factors for the development of cognitive impairment^14^. A study investigating the correlation between cognitive impairment and metabolic syndrome in schizophrenia revealed a significant association between MetS and cognitive impairment, suggesting that MetS might contribute to functional decline in individuals with schizophrenia^15^. However, there remains a gap in the literature concerning the examination of the relationship between MetS, cognitive impairment in CR-TRS patients.

Inflammation is widely acknowledged to underlie the pathophysiology of both schizophrenia and MetS, with cytokines playing a pivotal and indispensable role in these interconnected processes^16^. Previous evidence has identified elevated levels of pro-inflammatory cytokines in both the blood and cerebrospinal fluid (CSF) of patients with schizophrenia, including interleukin-6 (IL-6), and tumor necrosis factor-α (TNF-α)^17^. High levels of IL-6 have been associated with the severity of schizophrenia and the cognitive impairments^18^. Furthermore, pro-inflammatory cytokines (IL-6, TNF-α) have been demonstrated to be linked with MetS, as their concentrations were found to be elevated in MetS individuals^19^. Zhang et al. showed that IL-6 could serve as an effective marker for screening MetS in patients with chronic schizophrenia^20^. Although the association between MetS and inflammatory cytokines in schizophrenia patients has been reported, the relationship between MetS and cytokine levels in patients with CR-TRS has not been explored. The purpose of the study was to investigate the relationship between cytokines levels, clinical symptoms and cognitive impairments in CR-TRS patients, both with and without MetS. This goal was mainly addressed by: (1) comparing clinical symptoms, cognitive function and the levels of cytokines in CR-TRS patients with MetS and without MetS; (2) examining the relationship between cytokines levels, clinical symptoms and cognitive impairments in CR-TRS patients with and without MetS, respectively.

## Materials and methods

### Subjects

All subjects were enrolled from the Shanghai Pudong New Area Mental Health Center, between September 6, 2018, and August 1, 2021. The inclusion criteria were: (1) age between 18 and 65 years; (2) Han Chinese; (3) meeting diagnostic criteria for schizophrenia according to the Diagnostic and Statistical Manual of Mental Disorders, Fourth Edition (DSM-IV), using the Structured Clinical Interview for DSM-IV (SCID-I/P); (4) having undergone treatment with a minimum of two antipsychotic agents with different mechanisms, administered at appropriate doses for a sufficient course of treatment, and subsequently receiving a stable dose of clozapine (i.e., at least 400 mg/d or more for at least 6 months) to ensure a reasonable response to clozapine monotherapy; (5) a baseline PANSS score exceeding 60 before entering the study^21^. The exclusion criteria were: (1) any other major Axis I disorder; (2) serious physical diseases; (3) alcohol or other substance (other than tobacco) abuse/dependence; (4) pregnant or lactating women. A total of 84 healthy controls (HCs) were recruited without severe somatic diseases and personal or family history of psychiatric disorders.

This study was approved by the Institutional Review Board of Shanghai Pudong New Area Mental Health Center (No. 2018008), and written informed consent was obtained from each subject. The protocol was registered on clincialtrials.gov before participant enrollment (ID: NCT03652974).

### Clinical and cognitive assessment

The Positive and Negative Syndrome Scale (PANSS) was applied to assess psychopathological symptoms of patients^22^. In addition, the cognitive function of each participant was assessed using and the Repeatable Battery for the Assessment of Neuropsychological Status (RBANS)^23^. The RBANS is a widely utilized tool for neuropsychological assessment, encompassing five dimensions: attention, language, visuospatial/constructional, immediate memory, and delayed memory^24^. Additionally, repeated assessment showed that the inter-observer correlation coefficient exceeded 0.8.

### Laboratory measurement

Following an overnight fasting period, blood samples were obtained for standard blood analyses, encompassing measurements of fasting plasma glucose (FPG) as well as a comprehensive lipid profile. The lipid profile was assessed through determinations of triglyceride (TG), total cholesterol (TC), high-density lipoprotein cholesterol (HDL-C), and low-density lipoprotein cholesterol (LDL-C). All laboratory assessments adhered to the manufacturers’ stipulated protocols.

### Definition of MetS

The National Cholesterol Education Programme Adult Treatment Panel III (NCEP ATP III) criteria were used to define MetS^10^. Three or more of the following factors were categorized as MetS: (1) a waist circumference of ≥90 cm for men and ≥80 cm for women; (2) a fasting triglyceride level of ≥1.7 mmol/L; (3) a HDL-C level of <1.03 mmol/L for men and <1.30 mmol/L for women; (4) a systolic blood pressure of ≥130 mmHg or a diastolic blood pressure of ≥85 mmHg; (5) a FPG level of ≥5.6 mmol/L or reporting previously physician-diagnosed diabetes^25^.

### Cytokine determination

Venous blood samples were collected between 7 and 9 am from subjects following an overnight fast to assess cytokine levels, including IL-2, IL-6, and TNF-α. The collected blood samples underwent centrifugation to obtain serum, which was subsequently divided into aliquots and stored at -80 °C until required. The levels of cytokine were assayed by sandwich enzymelinked immunosorbent assay (ELISA) kit (Anogen, Mississauga, Ontario, Canada) according to the manufacturer’s instructions. Briefly, the standard or sample was added to the appropriate well of the antibody pre-coated microtiter plate and incubated. Then, the corresponding biotin was added and incubated, followed by 5 washes of the microtiter plate. Afterwards, the corresponding avidin was added, incubated and the wash procedure was repeated. Finally, substrate solution was added and incubated, followed by the addition of stop solution. According to the standard curve and Optical Density (O.D.) value of the tested sample, the concentration of cytokines in the sample was calculated.

### Statistical analysis

A Kolmogorov-Smirnov one-sample test was performed to assess the normal distribution of both the lipid profile and cytokine levels. In cases where normal distribution was not met, a logarithmic transformation was applied as a corrective measure. Categorical data were displayed in terms of frequencies, while quantitative data were represented as mean ± standard deviation (SD). Demographic and clinical characteristics, along with cognitive function and cytokine levels, were compared between groups using the chi-square test for categorical variables and analysis of variance (ANOVA) for quantitative variables. Additionally, gender, age, and BMI were selected as covariates in the analysis.

Receiver Operating Characteristic (ROC) curve analysis was employed to predict the differentiation between CR-TRS patients with and without MetS. Partial correlation analysis was conducted to explore the relationships between variables, while controlling for age, gender, and BMI. Additionally, the impact of cytokine profiles on clinical symptoms and cognitive functions was assessed using multiple linear regression analyses. All statistical analyses were performed using SPSS version 18.0. Visualization of differential expression and correlation analyses was accomplished using GraphPad Prism 6.0. A significance threshold of *p* < 0.05 was employed to determine statistical significance.

## Results

### Demographic, lipid profiles and clinical characteristics of subjects

A total of 69 CR-TRS patients and 84 HCs were recruited in this study. There were no significant differences in gender and education between CR-TRS patients and HCs (all *p* > 0.05). However, significant differences in gender and BMI were observed (*p* = 0.022 and *p* < 0.001, respectively), which were adjusted for in the subsequent analyses. Among the 69 CR-TRS patients, 31 (44.9%) were identified as having MetS. Compared with CR-TRS patients without MetS, CR-TRS those with MetS had a higher BMI (*F* = 16.559, *p* < 0.001). Moreover, we found that TG levels were significantly elevated in CR-TRS patients with MetS compared to those without MetS (*p* = 0.046), while no significant differences were noted in other aspects of the lipid profile (all *p* > 0.05) (Table 1).

**Table 1:** Demographic, lipid profiles and clinical characteristics of CR-TRS patients with MetS and without MetS and HCs.

Variables	Patients with MetS (*n* = 31)	Patients without MetS (*n* = 38)	HCs (*n* = 84)	*F*/χ^2^	*p* value
Age (years)	45.94 ± 9.63	48.68 ± 7.93	44.36 ± 7.21^#^	3.912	**0.022**
Gender (Male/ Female)	13/18	21/17	44/40	1.360	0.507
Education (years)	11.48 ± 2.43	11.21 ± 2.54	11.77 ± 2.64	0.652	0.523
BMI (kg/m^2^)	25.63 ± 3.71	22.85 ± 2.67^***^	22.15 ± 2.64^###^	16.559	**<0.001**
Lipid profiles					
FPG (mmol/L)	5.62 ± 1.88	5.19 ± 1.07	–	1.427	0.095
TG (mmol/L)	1.67 ± 0.67	1.35 ± 0.50	–	4.458	**0.046**
CHOL (mmol/L)	4.09 ± 0.79	4.08 ± 0.79	–	0.002	0.881
HDL-C (mmol/L)	1.14 ± 0.27	1.26 ± 0.31	–	2.875	0.056
LDL-C (mmol/L)	2.27 ± 0.80	2.23 ± 0.72	–	0.070	0.760
PANSS					
Total score	80.52 ± 7.47	82.05 ± 11.30	–	0.422	0.518
P subscore	19.87 ± 5.80	20.58 ± 5.63	–	0.263	0.610
N subscore	18.77 ± 5.33	20.24 ± 7.88	–	0.777	0.381
G subscore	41.55 ± 5.27	41.24 ± 6.47	–	0.047	0.830

Data presented as mean ± standard deviation or frequency.

Hashtag indicates significance between HCs and TRS patients. ^#^*p* < 0.05, ^###^*p* < 0.001.

Asterisk indicates significance between MetS and non-MetS groups. ^***^p < 0.01.

*CR-TRS* Clozapine-resistant treatment-refractory schizophrenia, *MetS* Metabolic Syndrome, *HCs* Healthy Controls, *BMI* Body Mass Index, *FPG* fasting plasma glucose, *TG* triglyceride, *CHOL* cholesterol, *HDL-C* high-density lipoprotein cholesterol, *LDL-C* low-density lipoprotein cholesterol, *PANSS* Positive and Negative Syndrome Scale, *P* positive symptom, *N* negative symptom, *G* general psychopathology syndrome.

Bold values identify statistical significance (*p* < 0.05).

### Cognitive function in CR-TRS patients with and without MetS along with HCs

In Table 2, we compared the cognitive function of 31 CR-TRS patients with MetS, 38 without MetS patients and 84 HCs, analyzing the RBANS total and five index scores. The results indicated significant differences among these three groups in both RBANS total score and the scores of the five dimensions (all *p* < 0.001). Compared with all CR-TRS patients, the HCs showed significantly higher RBANS total score and five index scores (all *p* < 0.001). Even after controlling for age, gender and BMI, there were still significant differences in the RBANS total score and these index scores (all *p* < 0.001).

**Table 2:** Differences in total and index scores of the RBANS between CR-TRS patients with MetS, without MetS, and HCs.

Cognition	Patients with MetS (*n* = 31)	Patients without MetS (*n* = 38)	HCs (*n* = 84)	*F*	*p* value	*p*^*a*^ value	*p*^*b*^ value
Immediate memory	54.45 ± 10.94	63.87 ± 10.04	74.54 ± 10.04	22.605	**<0.001**	**<0.001**	**<0.001**
Visuospatial/constructional	64.26 ± 11.78	71.45 ± 12.71	89.61 ± 17.97	36.972	**<0.001**	**<0.001**	**0.019**
Language	68.45 ± 15.51	73.39 ± 15.18	90.98 ± 12.35	40.319	**<0.001**	**<0.001**	0.125
Attention	70.68 ± 14.67	76.29 ± 12.26	94.45 ± 15.77	38.466	**<0.001**	**<0.001**	**0.007**
Delayed memory	55.55 ± 10.48	62.92 ± 10.78	79.73 ± 20.44	28.849	**<0.001**	**<0.001**	**0.007**
Total score	55.39 ± 8.30	62.74 ± 9.91	81.75 ± 16.83	50.807	**<0.001**	**<0.001**	**0.001**

Data presented as mean ± standard deviation.

*RBANS* Repeatable Battery for the Assessment of Neuropsychological Status, *CR-TRS* Clozapine-resistant treatment-refractory schizophrenia, *MetS* Metabolic Syndrome, *HCs* Healthy Controls.

^**a**^Indicates significance between HCs and CR-TRS patients, adjusted for age, gender, and body mass index (BMI).

^**b**^Indicates significance between MetS and non-MetS groups, adjusted for age, gender, and body mass index (BMI).

Bold values identify statistical significance (*p* < 0.05)

Furthermore, after adjusting for age, gender and BMI, CR-TRS patients with MetS had lower RBANS total score compared to those without MetS (*p* = 0.001). Similar results were found in the other four index scores, including immediate memory, visuospatial/constructional, attention and delayed memory (*p* < 0.001, *p* = 0.019, *p* = 0.007 and *p* = 0.007, respectively).

### Cytokine profiles in CR-TRS patients with and without MetS

**Fig. 1:**
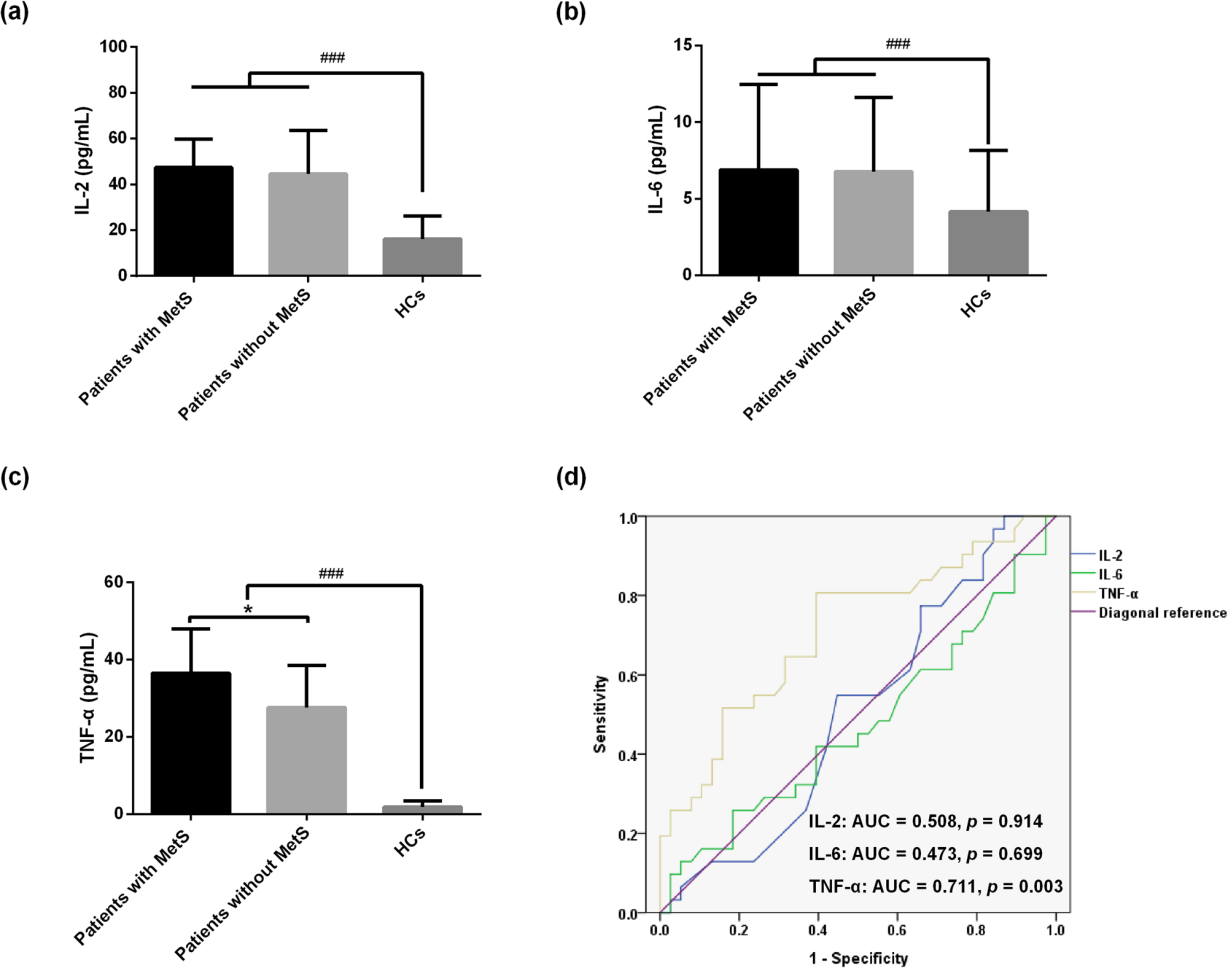
Cytokine profiles in CR-TRS patients with and without MetS. **a** Comparison of IL-2 between CR-TRS patients with MetS, without MetS, and HCs. **b** Comparison of IL-6 between CR-TRS patients with MetS, without MetS, and HCs. **c** Comparison of TNF-α between CR-TRS patients with MetS, without MetS, and HCs. Note: Hashtag indicates significance between HCs and CR-TRS patients. ^###^ *p* < 0.001. Asterisk indicates significance between MetS and non-MetS groups. ^*^ *p* < 0.05. **d** Receiver operating characteristic (ROC) curve of IL-2, IL-6 and TNF-α for predicting CR-TRS patients with and without MetS.

CR-TRS patients exhibited elevate the levels of IL-2, IL-6 and TNF-α compared with the HCs (all *p* < 0.001, Fig. 1). Interestingly, TNF-α levels were significantly higher in MetS patients than in CR-TRS patients without MetS (*p* < 0.05). These differences persisted even after controlling for age, gender and BMI (Supplementary Table 1). To further investigate the role of TNF-α in MetS, ROC curve analysis was conducted, revealing that TNF-α possessed a strong discriminatory capacity between CR-TRS patients with and without MetS (Fig. 1). The area under the curve (AUC) for this discrimination CR-TRS with and without MetS was 0.711 [95% confidence interval (CI) = 0.587–0.834].

### Relationship between cytokine and clinical variables, cognitive performance in CR-TRS patients with and without MetS

**Fig. 2:**
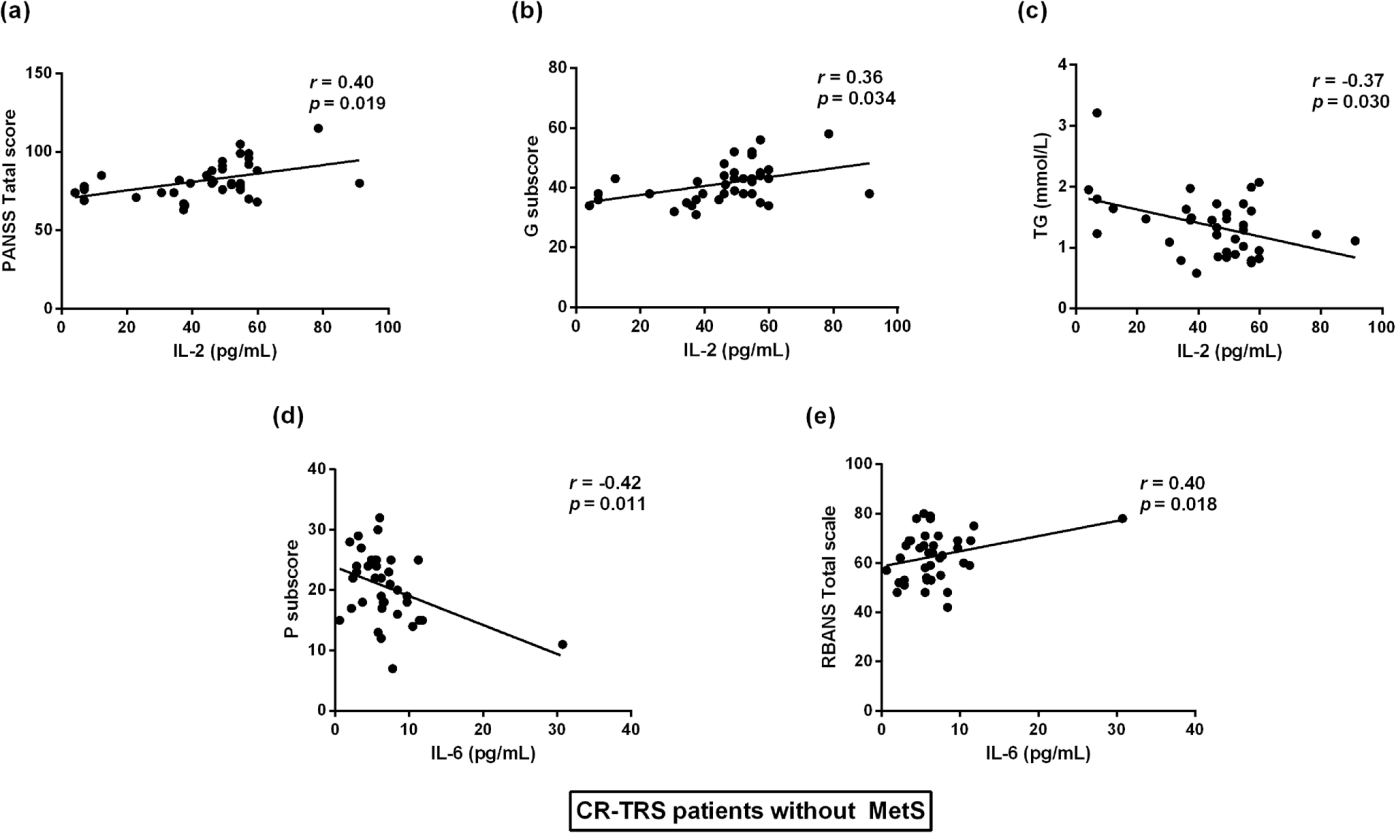
Correlation analysis between cytokine and clinical variables, cognitive performance measures or lipid profiles in CR-TRS patients without MetS. **a** Correlation analysis between the levels of IL-2 and PANSS total score. **b** Correlation analysis between the levels of IL-2 and G subscore of PANSS. **c** Correlation analysis between the levels of IL-2 and TG. **d** Correlation analysis between IL-6 levels and P subscore of PANSS. **e** Correlation analysis between IL-6 levels and the total score of RBANS. Note: Data were analyzed by the partial correlation analysis, after controlling for age, gender and body mass index (BMI). *p* values are significant (*p* < 0.05).

The partial correlation analysis was carried out to evaluate the relationship between cytokine and clinical variables, cognitive performance in CR-TRS patients with and without MetS, after controlling for age, gender and BMI (Supplementary Table 2). In CR-TRS patients without MetS, IL-2 levels were positively correlated with the PANSS total score and general psychopathology syndrome (G) subscore (*r* = 0.40, *p* = 0.019 and *r* = 0.36, *p* = 0.034, respectively, Fig. 2), while IL-2 levels exhibited a negative correlation with TG levels (*r* = –0.37, *p* = 0.030, Fig. 2).

Further stepwise multiple regression analysis identified IL-2 as an independent contributor to both the PANSS total score and G subscore (*β* = 0.454, *t* = 3.059, *p* = 0.004 and *β* = 0.437, *t* = 2.915, *p* = 0.006, respectively, Table 3). Additionally, IL-2 might be as an independent contributor to the positive symptom (P) subscore (*β* = 0.347, *t* = 2.220, *p* = 0.033, Table 3). Furthermore, IL-6 levels were significantly negatively correlated with the P subscore of PANSS (*r* = –0.42, *p* = 0.011, Fig. 2), and it was identified as an independent contributor to the P subscore of PANSS (*β* = -0.413, *t* = –2.723, *p* = 0.010, Table 3). In terms of cognition, IL-6 levels were positively correlated with the total score of RBANS (*r* = 0.40, *p* = 0.018, Fig. 2). Further stepwise multiple regression analysis identified IL-6 as an independent contributor to the delayed memory of RBANS (*β* = 0.404, *t* = 2.647, *p* = 0.012, Table 3).

**Table 3:** Stepwise regression models of PANSS total and subscore in CR-TRS patients without MetS^a^.

Outcome	Predictors	Patients without MetS (*n* = 38)		
		*β*	*t*	*p*
PANSS				
Total score	IL-2 (pg/mL)	0.454	3.059	**0.004**
P subscore	IL-2 (pg/mL)	0.347	2.220	**0.033**
G subscore	IL-2 (pg/mL)	0.437	2.915	**0.006**
P subscore	IL-6 (pg/mL)	–0.413	–2.723	**0.010**
RBANS				
Delayed memory	IL-6 (pg/mL)	0.404	2.647	**0.012**

*PANSS* Positive and Negative Syndrome Scale, *CR-TRS* Clozapine-resistant treatment-refractory schizophrenia, *MetS* Metabolic Syndrome, *P* positive symptom, *N* negative symptom, *G* general psychopathology syndrome, *RBANS* Repeatable Battery for the Assessment of Neuropsychological Status, *IL-2* interleukin-2, *IL-6* interleukin-6.

^a^After controlling for age, gender, body mass index (BMI).

## Discussion

To our knowledge, this study is the first to delve into the intricate relationship between cytokine levels, clinical symptoms, and cognitive impairments in CR-TRS patients, distinguishing between those with and without MetS. Our findings provide preliminary insights into the potential impact of cytokine levels within these subgroups. The main findings of the study were: (1) CR-TRS patients with MetS exhibited poorer cognitive function compared to those without MetS; (2) TNF-α levels were significantly higher in CR-TRS patients with MetS compared to those without MetS, showcasing promising pathophysiological potential for identifying CR-TRS patients with MetS; (3) in CR-TRS patients without MetS, IL-2 emerged as an independent contributor to the psychiatric symptom and general psychopathology subsymptoms. Additionally, IL-6 could be an independent contributor to the positive subsymptoms; (4) IL-6 was an independent contributor to delayed memory in CR-TRS patients without MetS.

In our study, we found that CR-TRS patients exhibited significantly lower scores on the RBANS total score and its five index scores compared to HCs. This contribution added to the growing body of research on the cognitive profiles of this special population. Previous studies have highlighted cognitive deficits in schizophrenia patients across various domains, including memory, attention, language, visuospatial abilities, processing speed, learning, and executive function^26^. Specifically, the TRS patients performed the most pronounced impairments in general neurocognition and social cognition^27^, while cognitive research on CR-TRS patients has been relatively limited. Furthermore, our study unveiled that even after controlling for age, gender and BMI, CR-TRS patients with MetS had lower cognition function than those without MetS, including immediate memory, visuospatial/constructional abilities, attention and delayed memory. These findings align with the majority of previous studies investigating cognitive function in schizophrenia patients with MetS. MetS has emerged as a crucial risk factor for the development of cognitive impairments in schizophrenia^28^. Several studies found that schizophrenia patients with MetS tend to perform poorly on specific cognitive tasks, including processing speed, attention/vigilance, working memory and problem solving/reasoning^29,30^. Additionally, a meta-analysis has affirmed a significant association between MetS and cognitive impairments in schizophrenia^15^. Collectively, these findings suggest that further study of the potential relationship between MetS and cognitive function in CR-TRS patients is warranted.

In recent years, mounting evidence underscores the pivotal role of cytokines, particularly pro-inflammatory ones, in the pathological progression of schizophrenia^31^. We demonstrated that CR-TRS patients exhibited elevated levels of IL-2, IL-6 and TNF-α in comparison to HCs, even after controlling for age, gender and BMI. These findings align with the prevailing trends observed in prior research on schizophrenia and MetS. For instance, previous research has demonstrated elevated inflammatory marker levels in individuals with both schizophrenia and MetS, as opposed to those without MetS^32^. Specifically, IL-2, a pro-inflammatory cytokine, displayed significantly elevated levels in individuals with chronic schizophrenia when compared to healthy subjects^33^. However, it’s worth noting that some studies have reported either decreased levels or non-significant differences in IL-2 levels among individuals with schizophrenia^17,34^. These discrepancies may arise from uncontrolled confounding factors or the variations in the selected populations of individuals with schizophrenia. Additionally, a study examining immuno-metabolic profiles identified five cytokines associated with MetS in schizophrenia, including IL-6 and TNF-α^35^. Zhang et al. reported that higher plasma IL-6 levels in schizophrenia patients treated with olanzapine or clozapine and with MetS compared to patients without MetS^36^. However, our study did not yield comparable findings for IL-6, potentially attributed to our specific focus on the CR-TRS population. Palmery et al. reported that TNF-α plays a significant role in inducing obesity-related insulin resistance, which is a key mechanism underlying MetS^37,38^. This underscores the importance of TNF-α in the pathophysiology of MetS. Notably, TNF-α has been identified as prognostic indicators for the emergence of MetS in patients with schizophrenia during antipsychotic treatment^39,40^. Our findings revealed markedly elevated TNF-α levels in CR-TRS patients with MetS compared to those without MetS, suggesting TNF-α‘s potential utility as a predictive marker for distinguishing between CR-TRS patients with and without MetS. These findings imply a potential involvement of heightened TNF-α levels in the context of MetS among CR-TRS patients, positioning it as a prospective pathophysiological biomarker for identifying MetS in this population.

Multiple observations have pointed to a potential link between cytokine concentrations and psychosis symptoms. In our study, we confirmed that IL-2 acted as an independent contributor to psychiatric symptom and general psychopathology subsymptoms in CR-TRS patients without MetS. However, similar positive associations were not detected in CR-TRS patients with MetS, and there was even an opposite trend. This discrepancy might arise from the increased complexity of CR-TRS patients with MetS and sample size, making such correlations less discernible. Another possible reason was that IL-2 might interact with certain inflammatory factors in the symptomatic mechanism of schizophrenia including a pathological mechanism of inflammatory damage from IL-2 compensation. It’s noteworthy that a study by Zhang et al. identified IL-2 as a significant factor influencing the cognitive and positive symptoms^33^. On the contrary, in a study exploring the cytokine profile (including IL-2, IL-10, IL-4, IL-6, IFN-γ, TNF-α, and IL-17) in patients experiencing their first episode of psychosis, no correlations were found between cytokine levels and the total PANSS score or the scores of the positive or negative subscales^41^. Notably, studies focusing on groups associated with the MetS were particularly scarce. Our results provided new clues and evidence regarding the role of IL-2 in CR-TRS patients, both with and without MetS.

Furthermore, out study revealed that IL-6 could be as an independent contributor to the positive subsymptoms in psychiatric symptoms. Recent research has consistently demonstrated that elevated serum concentrations of IL-6 were positively associated with severe clinical symptoms measured by the total, general, and negative scores of the PANSS scale in patients with schizophrenia^42^. A previous clinical trial also found that baseline total and general PANSS scores were positively correlated with the baseline levels of IL-6 in patients with schizophrenia^43^. Our results provide a more detailed understanding of the impact of IL-6 in CR-TRS patients. In terms of cognition, Donohoe et al. indicated the mediating role of IL-6 in early life adversity, functional connectivity and cognitive performance in schizophrenia^44^. High levels of IL-6 have been associated with the cognitive impairment suffered throughout schizophrenia^18^. Our study demonstrated a positive association between IL-6 levels and cognitive function, with IL-6 independently contributing to delayed memory in CR-TRS patients without MetS. These findings suggest that IL-6 might play a non-negligible role in psychiatric symptoms and cognition in CR-TRS patients without MetS.

There were several limitations in this study which should be concerned. Firstly, it was a cross-sectional study, and therefore, causality could not be established. Further prospective follow-up studies are necessary to elucidate causality. Secondly, the sample size of CR-TRS patients with or without MetS was relatively small. Larger sample sizes are required to validate and enhance the generalizability of the findings. Thirdly, only three cytokines, IL-2, IL-6 and TNF-α, were involved in this study, and the roles of many other cytokines remain unexamined. Future research should consider a broader cytokine profile to provide a more comprehensive understanding of the immune dysregulation in CR-TRS patients.

## Conclusions

In summary, our study provided initial insights into the potential interplay between cytokines levels, clinical symptoms and cognitive impairments in CR-TRS patients with and without MetS. Our findings suggested that TNF-α could potentially serve as a predictive marker for distinguishing between CR-TRS patients with and without MetS. Furthermore, IL-2 appeared to independently contribute to psychopathological symptoms in CR-TRS patients without MetS, while IL-6 appeared to independently contribute to cognitive function, particularly in the domain of delayed memory. Subsequent research involving multi-center studies with larger sample sizes and comprehensive multi-cytokine assays in CR-TRS patients will be valuable for a deeper exploration of the intricate relationships among cytokines, clinical symptoms, cognitive function, and MetS in the context of CR-TRS.

### Supplementary information

Supplementary Table 1. Comparison of cytokine levels between CR-TRS patients with MetS, without MetS, and HCs.

Supplementary Table 2. Correlations between cytokine and clinical variables, cognitive performance measures or lipid profiles in CR-TRS patients with MetS and without MetSa.

## Data Availability

The authors declare that all relevant data of this study are available within the article or from the corresponding author on reasonable request.
