# Supplementary material for: Association of cytokines levels, psychopathology and cognition among CR-TRS patients with metabolic syndrome
Source: Schizophrenia (Heidelb). 2024 Apr 16;10(1):47. doi: 10.1038/s41537-024-00469-x (PMC11021544; doi:10.1038/s41537-024-00469-x)
Supplement: Supplementary file 1 — Supplementary Table 1. Comparison of cytokine levels between CR-TRS patients with MetS, without MetS, and HCs. [file 41537_2024_469_MOESM1_ESM.docx]

**Supplementary Table 1**. Comparison of cytokine levels between CR-TRS patients with MetS, without MetS, and HCs.

| **Variable** | **Patients with MetS (n=31)** | **Patients without MetS (n=38)** | **HCs (n=84)** | ***F*** | ***p* value** | ***p^a^* value** | ***p^b^* value** |
| --- | --- | --- | --- | --- | --- | --- | --- |
| IL-2 (pg/mL) | 47.38±12.29 | 44.58±19.00 | 16.17±10.00 | 95.910 | **< 0.001** | **< 0.001** | 0.497 |
| IL-6 (pg/mL) | 6.89±5.57 | 6.77±4.83 | 4.16±4.01 | 6.463 | **0.002** | **0.001** | 0.336 |
| TNF-α (pg/mL) | 36.46±11.50 | 27.63±10.89 | 1.90±1.51 | 304.740 | **< 0.001** | **< 0.001** | **0.013** |

Data presented as mean± standard deviation.

**^a^** indicates significance between HCs and CR-TRS patients, adjusted for age, gender, and body mass index (BMI).

**^b^** indicates significance between MetS and non-MetS groups, adjusted for age, gender, and body mass index (BMI).

**Abbreviations:** CR-TRS, Clozapine-resistant treatment-refractory schizophrenia; MetS, Metabolic Syndrome; HCs, Healthy Controls; IL-2, interleukin-2; IL-6, interleukin-6; TNF-α, tumor necrosis factor-α.
