# Supplementary material for: Association of cytokines levels, psychopathology and cognition among CR-TRS patients with metabolic syndrome
Source: Schizophrenia (Heidelb). 2024 Apr 16;10(1):47. doi: 10.1038/s41537-024-00469-x (PMC11021544; doi:10.1038/s41537-024-00469-x)
Supplement: Supplementary file 2 — Supplementary Table 2. Correlations between cytokine and clinical variables, cognitive performance measures or lipid profiles in CR-TRS patients with MetS and without MetSa. [file 41537_2024_469_MOESM2_ESM.docx]

**Supplementary Table 2**. Correlations between cytokine and clinical variables, cognitive performance measures or lipid profiles in CR-TRS patients with MetS and without MetS**^a^**.

| **Variable** | **Patients with MetS (n=31)** | | | **Patients without MetS (n=38)** | | |
| --- | --- | --- | --- | --- | --- | --- |
|  | IL-2 (pg/mL) | IL-6 (pg/mL) | TNF-α (pg/mL) | IL-2 (pg/mL) | IL-6 (pg/mL) | TNF-α (pg/mL) |
| **PANSS** |  |  |  |  |  |  |
| Total score | -0.19 (0.333) | 0.01 (0.945) | 0.09 (0.646) | **0.40 (0.019)** | -0.13 (0.468) | 0.02 (0.917) |
| P subscore | 0.14 (0.489) | 0.22 (0.266) | 0.12 (0.543) | 0.28 (0.109) | **-0.42 (0.011)** | -0.26 (0.132) |
| N subscore | -0.37 (0.051) | -0.09 (0.668) | -0.16 (0.410) | 0.08 (0.659) | 0.28 (0.106) | 0.27 (0.123) |
| G subscore | -0.07 (0.708) | -0.05 (0.802) | 0.29 (0.137) | **0.36 (0.034)** | -0.22 (0.215) | -0.09 (0.623) |
| **RBANS** |  |  |  |  |  |  |
| Immediate memory | 0.14 (0.465) | -0.19 (0.335) | -0.24 (0.226) | 0.16 (0.362) | 0.30 (0.078) | 0.12 (0.486) |
| Visuospatial/  constructional | 0.13 (0.499) | -0.12 (0.537) | -0.01 (0.965) | 0.08 (0.665) | 0.04 (0.819) | 0.02 (0.891) |
| Language | 0.00 (1.000) | -0.28 (0.150) | 0.20 (0.297) | -0.01 (0.972) | 0.16 (0.351) | 0.25 (0.143) |
| Attention | 0.27 (0.170) | -0.37 (0.056) | 0.13 (0.510) | 0.13 (0.443) | 0.31 (0.070) | -0.10 (0.560) |
| Delayed memory | -0.00 (0.984) | -0.34 (0.077) | 0.11 (0.569) | -0.19 (0.333) | 0.01 (0.945) | 0.09 (0.646) |
| Total score | 0.10 (0.618) | -0.34 (0.079) | 0.08 (0.693) | 0.09 (0.610) | **0.40 (0.018)** | 0.08 (0.659) |
| **Lipid profiles** |  |  |  |  |  |  |
| FPG (mmol/L) | 0.05 (0.817) | -0.09 (0.659) | -0.08 (0.671) | -0.27 (0.113) | -0.06 (0.721) | 0.17 (0.332) |
| TG (mmol/L) | -0.03 (0.886) | 0.05 (0.816) | 0.06 (0.763) | **-0.37 (0.030)** | -0.15 (0.395) | 0.10 (0.578) |
| CHOL (mmol/L) | -0.03 (0.886) | 0.22 (0.252) | 0.16 (0.408) | -0.25 (0.146) | -0.09 (0.629) | 0.13 (0.468) |
| HDL-C (mmol/L) | -0.03 (0.864) | 0.02 (0.941) | 0.10 (0.600) | 0.15 (0.390) | 0.04 (0.839) | -0.29 (0.090) |
| LDL-C (mmol/L) | 0.01 (0.949) | 0.24 (0.211) | 0.15 (0.452) | -0.25 (0.149) | -0.08 (0.647) | 0.31 (0.068) |

Values were shown as *r* (*p*).

**^a^** Adjusting for age, gender, and body mass index (BMI).

**Abbreviations:** CR-TRS, Clozapine-resistant treatment-refractory schizophrenia; MetS, Metabolic Syndrome; IL-2, interleukin-2; IL-6, interleukin-6; TNF-α, tumor necrosis factor-α; PANSS, Positive and Negative Syndrome Scale; P, positive symptom; N, negative symptom; G, general psychopathology syndrome; RBANS, Repeatable Battery for the Assessment of Neuropsychological Status; FPG, fasting plasma glucose; TG, triglyceride; CHOL, cholesterol; HDL-C, high-density lipoprotein cholesterol; LDL-C, low-density lipoprotein cholesterol.
